# Oral Health and Social Isolation After 6 Years: Mediation of Oral Functions

**DOI:** 10.1111/cdoe.70035

**Published:** 2025-10-26

**Authors:** Hazem Abbas, Kenji Takeuchi, Taro Kusama, Sakura Kiuchi, Ken Osaka

**Affiliations:** ^1^ Department of International and Community Oral Health Tohoku University, Graduate School of Dentistry Sendai Japan; ^2^ Division of Statistics and Data Science, Liaison Center for Innovative Dentistry Tohoku University Graduate School of Dentistry Sendai Japan; ^3^ Frontier Research Institute for Interdisciplinary Sciences Tohoku University Sendai Japan

**Keywords:** cohort studies, dental prostheses, dentistry, epidemiology, geriatric dentistry, public health

## Abstract

**Objectives:**

Tooth loss was longitudinally associated with social isolation. The absence of dental prosthesis use was an additional risk factor. However, the mediating pathways for this association are unknown. The aim of this cohort study was to estimate the mediating effect of three oral functions: speaking, smiling and chewing observed at baseline on the association between oral health at baseline and social isolation after 6 years of follow‐up.

**Methods:**

The 2010–2016 panel data from 6103 functionally independent participants aged ≥ 65 years who were not socially isolated at baseline was used from the Japan Gerontological Evaluation Study (JAGES). Causal mediation analysis using parametric logistic regression models was used to calculate the natural direct effect (NDE), the natural indirect effect (NIE), and the total effect. Social isolation was derived from a 5‐point index and dichotomised (0/3 = not isolated, 4/5 = isolated). The confounders observed at baseline were age, sex, educational attainment, income, activities of daily living, living area, and having depressive symptoms assessed using the geriatric depression scale (GDS‐15).

**Results:**

The mean age of the participants was 72.4 years (SD = ±5.1), and 44.7% were males. Social isolation was observed at follow‐up among 3.6% of participants (*n* = 220). The cumulative incidence rate of socially isolated participants among those with ≥ 20 teeth was 2.9% increasing gradually to 3.2%, 3.5%, 3.7% and 7.2% among those with 10–19 teeth and used dental prosthesis, 10–19 teeth and did not use the dental prosthesis, 0–9 teeth and used dental prosthesis, and 0–9 teeth and did not use the dental prosthesis respectively. Compared with those with ≥ 20 teeth, having difficulty in speaking [NIE odds ratio (OR) = 1.02, 95% confidence interval (CI) = 0.93–1.11], problems in smiling (NIE OR = 1.02, 95% CI = 0.93–1.10) and difficulty in chewing (NIE OR = 1.04, 95% CI = 0.94–1.14) might have mediated the association for those with 10–19 teeth and used dental prosthesis as well as for those with 10–19 teeth without dental prosthesis (NIE OR = 1.02, 95% CI = 0.94–1.10), (NIE OR = 1.02, 95% CI = 0.93–1.11) and (NIE OR = 1.02, 95% CI = 0.93–1.11) respectively. While they might not have mediated the association for those with 0–9 teeth with and without dental prosthesis use.

**Conclusion:**

With low precision and uncertain estimates, limited oral function might have mediated the association between tooth loss (10–19 with and without dental prosthesis use groups) and social isolation over 6 years. It was uncertain whether a mediation effect of oral functions was observed for those with 0–9 teeth with and without dental prosthesis use.

## 1 Introduction

Social isolation is a pressing global health concern due to its severe negative effects on physical and mental well‐being [1, 2]. Previous studies have linked social isolation to increased all‐cause mortality and other adverse health outcomes, including cognitive decline, depression, dementia, higher risk of rehospitalisation, decreased resistance to infections, and a greater likelihood of falls among older adults [1, 3, 4, 5]. These health impacts are particularly pronounced in aging societies such as Japan and Korea [6, 7]. Given the projected increase in the global population aged 65 years or older to 1.5 billion by 2050 (16% of the world's population), social isolation is expected to become a significant global health burden [8]. Recently, the World Health Organization (WHO) commission on social connection recommended community and individual‐level strategies to tackle social isolation and loneliness [2]. The commission advocates for integrating social connection strategies into policies, urban planning, and health systems, while also investing in research and public awareness to address the issue [2].

A decline in oral health status was associated with social isolation among the older population in the Japanese, English, and Brazilian contexts [9, 10, 11]. A previous longitudinal study from Japan using the same waves of the dataset used for this study showed that a fewer number of remaining teeth was associated with social isolation after 6 years of follow‐up, and no dental prosthesis use was an additional risk factor [9]. However, to date, the mechanism for this association was unknown. Previous studies suggested that tooth loss and not using dental prosthesis have a negative effect on conversational ability (speaking) [12], self‐esteem, and facial attractiveness (smiling with confidence) [13, 14], and subsequently, it might contribute to withdrawal from the surrounding society and eventually lead to social isolation [9, 10]. Additionally, tooth loss compromises chewing abilities, influences food intake [15], and quality of nutrients [16] leading to malnutrition, which was associated with underweight and frailty, a precursor for social isolation among the older population [9, 10, 17, 18].

The aim of this study was to examine the effect of three oral functions (speaking, smiling, and chewing) observed at baseline as mediators between oral health status represented by the number of teeth and dental prosthesis use at baseline and social isolation at follow‐up in an older Japanese population.

## 2 Methods

In this cohort study, the 2010–2016 panel data collected through a postal survey from the Japan Gerontological Evaluation Study (JAGES) was used. The JAGES is an ongoing study targeting functionally independent community‐dwelling adults aged 65 years or older to study their social, behavioural, and health‐related factors. Between 2010 and 2016, a combination of simple random sampling and complete survey for all 65 years or older residents was conducted alternatively in 13 municipalities across Japan [19]. The JAGES has a core questionnaire distributed to all eligible targeted participants and a set of subsidiary questionnaires distributed randomly to one in four of the eligible targeted participants. The questions related to the deterioration of the three oral functions used as mediators in this study were part of the subsidiary questionnaire. The inclusion criteria for this study were answering the subsidiary questionnaire, being functionally independent, and not socially isolated at baseline to have an outcome‐free cohort.

### 2.1 Exposure Variables (Independent Variables)

Based on previous studies, self‐reported number of remaining teeth and dental prosthesis use at baseline represented oral health status [9, 10]. The question “How many natural teeth do you have in your mouth?” was used to identify the number of remaining teeth. Its four responses were “20 or more teeth”, “10–19 teeth”, “1–9 teeth” and “no natural teeth”. However, the last two categories were combined into the “0–9 teeth” category due to the small proportions of these participants. While the question “Do you wear dentures or bridges (non‐removable dentures)?” was used to determine dental prosthesis use. Its four responses were “No”, “Yes, in the upper jaw”, “Yes, in the lower jaw” and “Yes, in both jaws”. These answers were dichotomised into (dental prothesis use vs no dental prosthesis use). Then, based on previous studies, these two variables were combined to derive the main exposure variable with its five categories: “≥ 20 natural teeth”, “10–19 natural with dental prosthesis”, “10–19 natural teeth without dental prosthesis”, “0–9 natural teeth with dental prosthesis” and “0–9 natural teeth without dental prosthesis” [9, 10]. In the sensitivity analyses, number of remaining teeth and dental prosthesis use were used as two separate exposures in separate analytical models. Self‐reported number of teeth of the older adults in Japan was previously validated [20, 21].

### 2.2 Outcome Variable (Dependent Variable)

Social isolation is an objective multidimensional concept that encompasses withdrawal from the surrounding society in the form of lack of social contacts and interactions with family members, friends, and the surrounding community [22, 23, 24]. However, there is no consensus on a clear definition of social isolation [25]. In this study, an objective comprehensive multidimensional variable was used to measure social isolation [9, 26, 27]. A 5‐point index was derived from the binary responses to the following five domains of questions collected at follow‐up in 2016: (1) being married or cohabitating with a partner; (2) living with one's own children or grandchildren or having someone to provide emotional or instrumental social support; (3) having immediate family members or relatives who could provide emotional or instrumental social support; (4) having face‐to‐face contact with friends more than once a month or having friends who could provide emotional or instrumental social support; and (5) participation in any volunteer group, leisure activity group, senior citizen club, neighbourhood or residents' association, and industrial or trade association [24, 27]. A score of zero indicated no social isolation, and a score of five indicated severe social isolation. Based on previous studies and using a data‐driven approach, a binary variable was derived from this 5‐categorical variable as follows: Scores 0 to 3 were not socially isolated, and scores 4 and 5 were socially isolated [9, 10]. The predictive and convergent validities of the social isolation index have been confirmed in previous studies [10, 28, 29].

### 2.3 Mediators

The deteriorations in three oral functions observed at baseline were used as hypothesised mediators for the association between oral health status and social isolation. They were having difficulty in speaking, having problems in smiling, and having difficulty in chewing.

Two questions derived from the short version of the Oral Impacts on Daily Performance (OIDP) questions in the JAGES subsidiary questionnaire were used. Participants were asked, “Have you had any problems related to your teeth, gums, and/or dentures during the past 6 months?” and were able to choose a binary answer (yes/no) regarding the following two items: “Difficulty in speaking clearly” and “Hesitated to show teeth when laughing or talking”. While for having difficulty in chewing, the following question regarding subjective chewing ability was used: “How well can you eat hard food?” and its five categorical answers were “I can chew and eat anything I want.”, “I have trouble chewing some foods, but I can eat most foods.”, “I can't chew well, and can only eat limited foods.”, “I can hardly chew at all.” and “I cannot chew at all, and I am on a liquid diet.” Those who chose one of the first two answers were categorised as not having difficulty in chewing, while those who chose one of the last three answers were categorised as having difficulty in chewing.

### 2.4 Confounders

**FIGURE 1:**
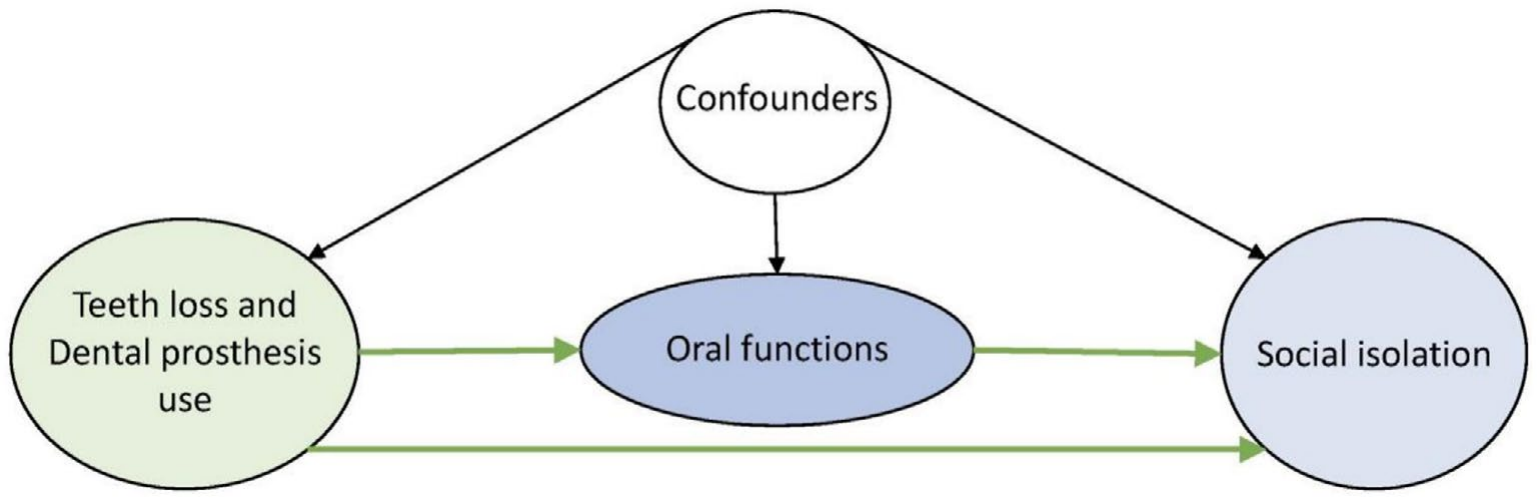
The hypothesised directed acyclic graph (DAG) for the mediation analysis of the association between the number of remaining teeth and dental prosthesis use with social isolation. Confounders were age, sex, educational attainment, equivalised income level, independent ability of daily living, area of residence and depressive symptoms. The three mediating oral functions were difficulty in speaking, problems in smiling and difficulty in chewing.

A directed acyclic graph (DAGitty version 3.0) was used to structure the theoretical framework of this study (Figure 1). Based on previous studies and by following a conservative approach for selecting the confounders [9], the data collected at the baseline in 2010 for demographics, socioeconomic status (SES), health and lifestyle, and area of residence were included as confounders. Age and sex were included as demographic confounders. Educational attainment expressed by years of formal education and the equivalised household income (i.e., annual pretax household income/square root of the number of family members) were included as SES confounders [30]. The reported income in Japanese Yen (JPY) was converted to United States Dollar (USD) at the common average rate of (100 JPY = 1 USD) at the time of data collection in 2010. For health and lifestyle, the 13‐question version of instrumental activities of daily living (IADL) was used. One point of the score was assigned to each question. A score of zero indicated complete dependence on others for daily‐life activities, and a score of 13 indicates complete independence [31]. The IADL score was dichotomised into dependent (IADL score = 0–12) and independent (IADL score = 13). Area of residence was determined by calculating population density based on 2010 national census data and was categorised into three categories: urban (≥ 4000 people/km^2^), suburban (1000–3999 people/km^2^), and rural (0–999 people/km^2^) [32]. Having depressive symptoms was assessed using the geriatric depression scale (GDS‐15) and was included as a psychological confounder [33]. Using the cut‐off value (GDS score = 5) as suggested by previous studies, the GDS‐15 scores were dichotomised to indicate having depression symptoms [33, 34].

### 2.5 Statistical Analysis

Descriptive analyses examined the baseline characteristics of the sample as well as the cumulative incidence rate of social isolation at follow‐up. Causal mediation analysis using paramed command with logistic regression was used to calculate the odds ratios (ORs) and 95% confidence intervals (95% CIs) of the natural direct effect (NDE), the natural indirect effect (NIE), and the total effect (TE) for the deterioration of the three oral functions on the association between oral health and social isolation [35]. The proportion mediated (PM) was also calculated as it indicated the proportion of TE through a given mediator. Causal mediation analyses address biases in the classical and traditional methods (product or difference methods) by using the counterfactual framework allowing for exposure–mediator interaction through calculating the direct and indirect effects [35]. Paramed command in Stata accommodates binary variables. The exposure was a five‐categorical variable; consequently, four datasets were created to allow for analysis of each oral health category in comparison to the reference (≥ 20 teeth); thus, the analyses were conducted four times for each mediator. In addition, dummy variables for the confounders were created. Multiple imputation method with chained equations was used to address potential biases due to missing data in the panel dataset.

For sensitivity analyses, first, the two variables representing oral health (number of teeth and dental prosthesis use) were used as two separate exposures in supplementary causal mediation analyses. Second, to assess the effect of the unmeasured confounders, the E‐values were calculated. The E‐value represents the minimum strength of association that an unmeasured confounder would need to have with both the exposure and outcome to explain away the observed association conditional on the measured confounders [36]. Third, logistic regression was used to calculate the odds ratio for the incidence of social isolation at follow‐up using a combined variable for the number of remaining teeth and dental prosthesis use in two regression models, a crude model and a fully adjusted model. Fourth, the interaction and effect modification between dental prosthesis use and the number of remaining teeth on social isolation were also examined. Fifth, the relationship between oral health and social isolation was examined using the inverse probability of treatment weighting (IPTW) method with multinomial regression to reduce bias resulting from the heterogeneity in the distribution of measured confounders among the participants by creating a pseudo‐population where the treatment assignment was independent of the observed confounders through applying weights based on the inverse of the propensity score. Sixth, the descriptive and the logistic regression data analyses using the complete data without multiple imputation were reported in the Tables S1–S9. Stata/SE 16 software from StataCorp LP (College Station, Texas, USA) was used for statistical analyses, and the STROBE guidelines for cohort studies were followed. No content of this study was created using Artificial Intelligence (AI).

### 2.6 Ethics Statement

Ethical approval for the JAGES project 2010 was obtained from the Ethics Committee of Nihon Fukushi University (approval number: 10‐05). Ethical approval for the study of the JAGES project 2016 was obtained from the Ethics Committee of the National Center for Geriatrics and Gerontology (approval number: 992) and Chiba University (approval number: 2493). Participants were notified that participation was voluntary, and returning an answered survey was considered a consent to participation.

## 3 Results

**FIGURE 2:**
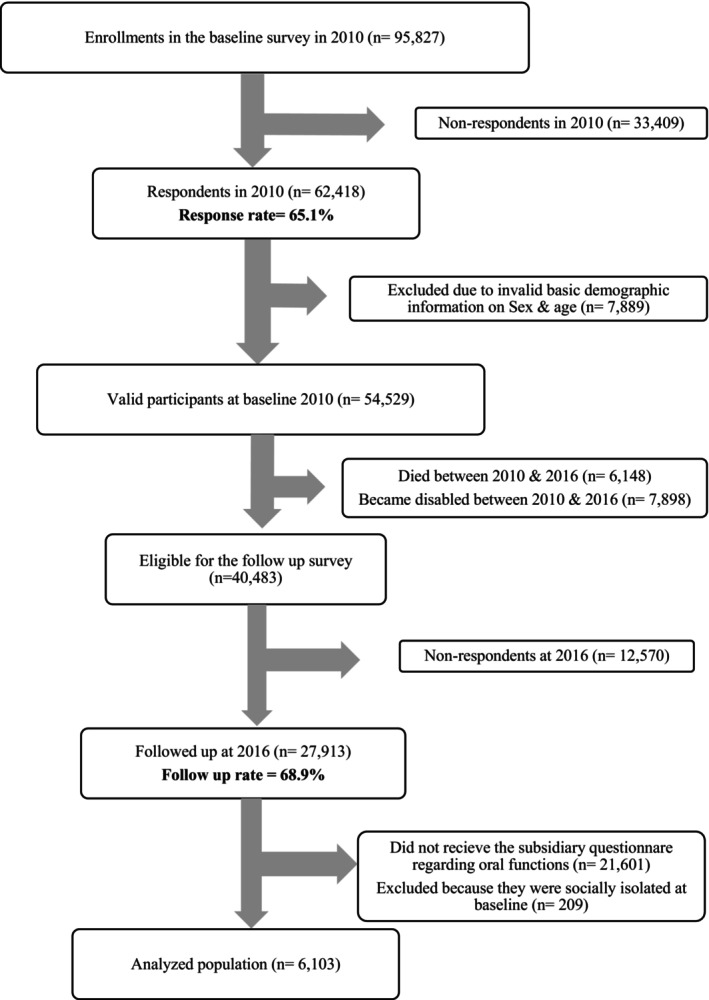
Flow chart of the participants from baseline in 2010 to follow‐up in 2016.

Figure 2 shows the flow chart of the study participants. The total number of participants who replied to both waves of the postal survey was 27 913 participants, with a follow‐up rate of 68.9%. In total, 6103 participants were eligible as study participants according to the inclusion criteria. Table 1 shows the baseline characteristics of the participants according to their oral health. The mean age of the study participants was 72.4 years (SD = ±5.1), and 44.7% were males. Social isolation was observed at follow‐up in 3.6% of participants (*n* = 220). While Table 2 showed the breakdown of the cumulative incidence rate of social isolation status at the follow‐up as follows: 71 participants (2.9%) had ≥ 20 teeth (with or without using dental prosthesis), 34 (3.2%) had 10–19 teeth and used dental prosthesis, 21 (3.5%) had 10–19 teeth and did not use the dental prosthesis, 54 (3.7%) had 0–9 teeth and used dental prosthesis, and 41 participants (7.2%) had 0–9 teeth and did not use the dental prosthesis. The cumulative incidence of social isolation was higher among those who had difficulty in speaking, problems in smiling, and difficulty in chewing. Those with fewer teeth and no dental prosthesis use, males, older age groups, less educated, lower income groups, dependent on others for their daily life activities, living in urban areas, and those who had depressive symptoms showed higher cumulative incidence rates of being socially isolated at follow‐up. Tables S1 and S2 showed the baseline characteristics of the study participants and the proportions of social isolation at follow‐up before multiple imputation. The findings of ST1 and ST2 showed similar pattern direction and magnitude compared with the main analyses.

**TABLE 1:** Baseline characteristics of the participants by oral health status after multiple imputation (*n* = 6103).

	Total		≥ 20 teeth		10–19 teeth with dental prosthesis		10–19 teeth without dental prosthesis		0–9 teeth with dental prosthesis		0–9 teeth without dental prosthesis	
	No.	%	No.	%	No.	%	No.	%	No.	%	No.	%
**Difficulty in speaking**												
No	5756	94.3	2425	42.1	977	17.0	577	10.0	1276	22.2	501	8.7
Yes	347	5.7	30	8.6	73	21.0	19	5.5	156	44.9	70	20.0
**Problems in smiling**												
No	5743	94.1	2413	42.0	976	17.0	565	9.8	1286	22.4	504	8.8
Yes	360	5.9	42	11.6	74	20.5	32	8.8	146	40.5	67	18.5
**Difficulty in chewing**												
No	5692	93.3	2434	42.8	992	17.4	566	9.9	1245	21.9	456	8.0
Yes	411	6.7	21	5.2	58	14.0	31	7.5	187	45.4	115	28.0
**Age**												
65–69 years	2115	34.7	1014	47.9	395	18.7	210	9.9	370	17.5	127	6.0
70–74 years	2104	34.5	881	41.9	368	17.5	211	10.0	466	22.1	178	8.5
75–79 years	1249	20.5	417	33.4	189	15.1	131	10.5	343	27.4	170	13.6
80–84 years	510	8.4	125	24.4	86	16.8	36	7.1	188	36.9	76	14.9
≥ 85 years	125	2.0	19	15.2	12	9.9	8	6.5	65	52.1	20	16.2
**Sex**												
Male	2727	44.7	1112	40.8	494	18.1	252	9.3	624	22.9	245	9.0
Female	3376	55.3	1343	39.8	556	16.5	344	10.2	807	23.9	326	9.7
**Years of formal education**												
≥ 13 years	1126	18.4	573	50.9	212	18.8	76	6.8	212	18.8	53	4.7
10–12 years	2251	36.9	962	42.7	420	18.7	226	10.0	500	22.2	144	6.4
≤ 9 years	2726	44.7	921	33.8	418	15.3	294	10.8	720	26.4	374	13.7
**Equivalised income level**												
< 20 000 USD/Year	2848	46.7	1002	35.2	440	15.4	309	10.8	729	25.6	368	12.9
20 000–39 999 USD/Year	2568	42.1	1111	43.2	483	18.8	243	9.5	565	22.0	167	6.5
> 40 000 USD/Year	687	11.2	342	49.9	127	18.5	44	6.5	137	20.0	36	5.2
**Independence in activities of daily living (IADL)**												
IADL score 0–12 (dependent)	3036	49.7	1141	37.6	493	16.2	322	10.6	750	24.7	330	10.9
IADL score 13 (independent)	3067	50.3	1314	42.8	557	18.2	274	8.9	681	22.2	241	7.8
**Living area**												
Urban	1211	19.8	570	47.1	212	17.5	114	9.4	248	20.5	66	5.5
Sub‐urban	2424	39.7	1030	42.5	426	17.6	224	9.2	550	22.7	195	8.0
Rural	2468	40.4	856	34.7	411	16.7	258	10.4	633	25.7	310	12.6
**Depressive symptoms (GDS‐15)**												
No (0–4)	4699	77.0	1968	41.9	822	17.5	445	9.5	1056	22.5	407	8.7
Yes (≥ 5)	1404	23.0	487	34.7	227	16.2	151	10.8	375	26.7	164	11.7
**Total**	6103	100.0	2455	40.2	1050	17.2	596	9.8	1431	23.5	571	9.4

Abbreviation: GDS = geriatric depression scale.

**TABLE 2:** The cumulative incidence rate of social isolation at follow‐up after multiple imputation (*n* = 6103).

	Total		Not isolated		Isolated	
	No.	%	No.	%	No.	%
**Oral health status**						
≥ 20 teeth	2455	40.2	2384	97.1	71	2.9
10–19 teeth with dental prosthesis	1050	17.2	1016	96.8	34	3.2
10–19 teeth without dental prosthesis	596	9.8	575	96.5	21	3.5
0–9 teeth with dental prosthesis	1431	23.5	1378	96.3	54	3.7
0–9 teeth without dental prosthesis	571	9.4	530	92.8	41	7.2
**Difficulty in speaking**						
No	5756	94.3	5550	96.4	206	3.6
Yes	347	5.7	333	95.9	14	4.1
**Problems in smiling**						
No	5743	94.1	5539	96.5	204	3.5
Yes	360	5.9	344	95.5	16	4.5
**Difficulty in chewing**						
No	5692	93.3	5502	96.7	190	3.3
Yes	411	6.7	381	92.7	30	7.3
**Age**						
65–69 years	2115	34.7	2054	97.1	61	2.9
70–74 years	2104	34.5	2037	96.8	67	3.2
75–79 years	1249	20.5	1192	95.4	57	4.6
80–84 years	510	8.4	484	94.8	26	5.2
≥ 85 years	125	2.0	117	93.4	8	6.6
**Sex**						
Male	2727	44.7	2599	95.3	128	4.7
Female	3376	55.3	3284	97.3	92	2.7
**Years of formal education**						
≥ 13 years	1126	18.4	1093	97.1	33	2.9
10–12 years	2251	36.9	2173	96.5	78	3.5
≤ 9 years	2726	44.7	2617	96	110	4
**Equivalised income level**						
< 20 000 USD/Year	2848	46.7	2715	95.3	133	4.7
20 000–39 999 USD/Year	2568	42.1	2492	97	77	3
> 40 000 USD/Year	687	11.2	677	98.6	10	1.4
**Independence in activities of daily living (IADL)**						
IADL score 0–12 (dependent)	3036	49.7	2860	94.2	176	5.8
IADL score 13 (independent)	3067	50.3	3023	98.6	44	1.4
**Living area**						
Urban	1211	19.8	1149	94.9	62	5.1
Sub‐urban	2424	39.7	2348	96.9	76	3.1
Rural	2468	40.4	2387	96.7	81	3.3
**Depressive symptoms (GDS‐15)**						
No (0–4)	4699	77.0	4571	97.3	127	2.7
Yes (≥ 5)	1404	23.0	1312	93.4	93	6.6
**Total**	6103	100.0	5883	96.4	220	3.6

Abbreviation: GDS = Geriatric depression scale.

Table 3 shows the findings of the causal mediation analyses. Considering the observed wide (95% CIs) of most of the NDE and TE, the findings did not reject the null hypothesis; however, the TE of oral health status on social isolation tended to be higher among those with worse oral health status in all mediation models of the three oral functions. In addition, compared with those with 20 or more teeth, having difficulty in speaking (NIE OR = 1.02, 95% CI = 0.93–1.11), problems in smiling (NIE OR = 1.02, 95% CI = 0.93–1.10), and difficulty in chewing (NIE OR = 1.04, 95% CI = 0.94–1.14) might have mediated the association between tooth loss at baseline and social isolation at follow‐up for those with 10–19 teeth and used dental prosthesis as well as for those with 10–19 teeth without dental prosthesis (NIE OR = 1.02, 95% CI = 0.94–1.10), (NIE OR = 1.02, 95% CI = 0.93–1.11), and (NIE OR = 1.02, 95% CI = 0.93–1.11), respectively. While they might not have mediated the association for those with 0–9 teeth with and without dental prosthesis, the proportion mediated through having difficulty in chewing was higher than the other two oral functions among those having 10–19 teeth with and without dental prosthesis. Overall, these three mediators explained 18%–51% of the relationship between oral health and social isolation for those with 10–19 teeth and used dental prosthesis, while this proportion was 14.8%–15.7% for those with 10–19 teeth and did not use dental prosthesis.

**TABLE 3:** The mediating effect of oral function decline in the association between oral health status and social isolation after multiple imputation (*n* = 6103).

Number of remaining teeth (ref. ≥ 20)	Mediators								
	Difficulty in speaking			Problems in smiling			Difficulty in chewing		
	OR	(95% CI)		OR	(95% CI)		OR	(95% CI)	
**10–19 with dental prosthesis**									
Natural direct effect	1.09	0.56	1.61	1.10	0.56	1.65	1.04	0.51	1.57
Natural indirect effect	1.02	0.93	1.11	1.02	0.93	1.10	1.04	0.94	1.14
Total effect	1.11	0.59	1.63	1.12	0.59	1.65	1.08	0.56	1.61
Proportion mediated	19.5%			18.0%			51.0%		
**10–19 without dental prosthesis**									
Natural direct effect	1.13	0.33	1.94	1.13	0.34	1.93	1.12	0.34	1.90
Natural indirect effect	1.02	0.94	1.10	1.02	0.93	1.11	1.02	0.93	1.11
Total effect	1.15	0.34	1.96	1.16	0.35	1.96	1.15	0.35	1.94
Proportion mediated	14.8%			14.8%			15.7%		
**0–9 with dental prosthesis**									
Natural direct effect	1.18	0.70	1.67	1.16	0.69	1.63	1.17	0.68	1.66
Natural indirect effect	0.99	0.91	1.07	1.00	0.93	1.07	1.00	0.91	1.09
Total effect	1.17	0.70	1.64	1.16	0.69	1.62	1.17	0.70	1.64
Proportion mediated	—			—			—		
**0–9 without dental prosthesis**									
Natural direct effect	2.41	1.36	3.46	2.19	1.16	3.21	2.49	1.57	3.41
Natural indirect effect	0.94	0.87	1.01	1.01	0.90	1.12	0.94	0.83	1.06
Total effect	2.27	1.31	3.23	2.20	1.26	3.14	2.34	1.45	3.24
Proportion mediated	—			1.8%			—		

*Note:* Adjusted for age, sex, educational attainment, income, activities of daily living, living area, and having depressive symptoms.

Abbreviations: 95% CI, 95% confidence intervals; OR, odds ratio.

In the sensitivity analyses, a similar pattern, direction, and magnitude of the mediation findings were observed. Table S3 shows the mediating effect of oral functions decline when only the number of teeth was used as an exposure. Oral functions might have mediated the association for those with 10–19 teeth but might not have for those with 0–9 teeth. While Table S4 showed the findings when only dental prosthesis use was used as an exposure, oral functions might not have mediated the association in this model. Table S5 shows the estimated E‐values. E‐values' estimates were relatively large and varied from 1.37 to 4.11 in odds ratio scales, suggesting robust estimates of this study even if there were unmeasured confounders. Tables S6 and S7 show the estimates of the association between oral health (using a combined variable for the number of remaining teeth and dental prosthesis use) and social isolation using logistic regression on both the imputed data and the complete case scenarios. A similar pattern, direction, and magnitude of the estimates were observed in both scenarios. Table S8 showed the interaction and effect modification between dental prosthesis use and the number of remaining teeth on social isolation. The findings were contrasting depending on the number of remaining teeth. Dental prosthesis use seemed to mitigate the odds of incidence of social isolation at follow‐up for those with 0–9 remaining teeth (i.e., synergistic effect) but not for those with 10–19 remaining teeth (i.e., antagonistic effect) using both the interaction additive and multiplicative scales. Table S9 showed the findings of the relationship between oral health and social isolation at follow‐up using IPTW. A pattern similar in direction but considerably smaller in magnitude compared to the findings of the logistic regression models was observed (i.e., in the adjusted model, the group having 0–9 teeth without dental prosthesis showed a considerably small (2%) likelihood to be socially isolated at follow‐up compared to the group with 20 or more teeth).

## 4 Discussion

To the best of our knowledge, this was the first study to investigate the mediation effect of three oral functions (speaking, smiling, and chewing) on the association between oral health and social isolation. Considering the low precise and high uncertain estimates, the findings did not reject the null hypothesis; however, having difficulty in speaking, problems in smiling, and difficulty in chewing might have mediated the association between oral health status and social isolation among those having 10–19 teeth with and without dental prosthesis but might not have among those having 0–9 teeth with and without dental prosthesis. The proportions mediated through having difficulty in chewing were higher than those of the other two oral functions among those having 10–19 teeth with and without dental prosthesis.

These findings were consistent with the hypothesised mechanisms explaining the association between oral health and social isolation in previous studies [9, 10]. Tooth loss and not using dental prosthesis had adverse effects on conversational ability (speaking) [12], self‐esteem, and facial attractiveness (smiling with confidence) [13, 14], and subsequently, it might contribute to withdrawal from the surrounding society and eventually lead to social isolation [9, 10]. In addition, compromised chewing abilities resulting from tooth loss influence food intake [15], and quality of nutrients [16] leading to malnutrition, which was associated with underweight and frailty, a precursor for social isolation among the older population [9, 10, 17, 18].

To explain the variation in the findings among oral health groups, those with 10–19 teeth with and without dental prosthesis use (moderate oral health deterioration group) tended to be younger, more educated, wealthier, more independent, and had fewer depressive symptoms. Thus, it's highly likely that their communication ability, facial appearance, and eating ability were of major importance, and the decline of these oral functions had a higher impact on them and led to their social isolation. On the contrary, those with 0–9 teeth with and without dental prosthesis use (severe oral health deterioration group) tended to be older, less educated, less wealthy, dependent on others for their daily activities, living in rural areas, and had more depressive symptoms. Thus, it's highly likely that their communication ability, facial appearance, and eating ability were of minor importance, and non‐oral health‐related mediators such as depression, social anxiety, chronic stress, reduced self‐esteem, and unhealthy diet had a higher impact on them and led to their social isolation status. According to previous studies, more tooth loss was associated with having more depressive symptoms [37] and reduced self‐esteem [13], and having depressive symptoms was associated with social isolation [38]. Thus, depression resulting from tooth loss, especially in the case of anterior teeth loss among other psychological factors, might mediate the association between oral health and social isolation, especially for those with severe tooth loss (0–9 teeth groups).

The findings of this study emphasised the importance of social functions of oral health as it showed that the decline in speaking and smiling as chief communication tools could be a pathway to future social isolation, a relatively less emphasised importance in the literature [9] compared to the already emphasised importance of the decline in eating abilities (in the form of compromised chewing ability) as the nutritional function of oral health [39]. Interventions aiming for retention of natural teeth and the provision of dental prosthesis might help reduce the oral health‐related burden on social isolation.

The findings of this study should be interpreted with caution considering its limitations. First, the wide confidence intervals observed suggested high uncertainty and low precision of the estimates. This could be attributed to the smaller sample size compared to the previous study (almost 1/5 of the sample of the previous study) as the mediators' questions were part of the subsidiary, not the core questionnaire of JAGES [9]. This calls for caution in interpreting the findings and potentially for further research with a larger sample size to help narrow the confidence interval and provide a more precise estimate of the effect. Second, despite the conservative approach to adjust for potential confounders, the possibility of the existence of unmeasured confounders could not be ruled out. However, E‐values were calculated to assess the magnitude of the unmeasured confounders, and their findings showed that the magnitude of unmeasured confounders was relatively small. Third, although the three included oral functions mediated between 14.8% and 51% of the association between oral health and social isolation, the role of other oral health‐related mediators, such as dental pain and discomfort, could not be estimated due to data limitations. Fourth, selection bias due to dropouts during the 6 years of follow‐up might have led to the underestimation of the findings. Those who dropped out as non‐respondents were 12 570 participants; other dropouts died or became disabled (Follow‐up rate = 68.9%). Dropouts are usually considered to have worse health and oral health statuses. Thus, if dropouts were included, the findings might have been larger.

Fifth, time‐varying confounding such as income fluctuations, changes in independence in activities of daily living (IADL), changes in living area, and having depressive symptoms over the 6 years of follow‐up may have influenced the findings. However, the income data showed similar income levels at follow‐up (mean 35 800, standard deviation 28 000 and median 27 500 USD/Year) compared to baseline (mean 39 600, standard deviation 28 100 and median 35 000 USD/Year), and the baseline equivalised income was adjusted in the analyses, in addition to the fact that the participants of this study were aged 65 years and older and their main source of income tended to be their pension; thus, income fluctuations within this age group are believed to be low in Japan [40, 41]. Similarly, the proportions of those living in urban, suburban, and rural areas as well as for those having versus not having depressive symptoms were similar at follow‐up compared to baseline. However, the changes in independence in activities of daily living (IADL) between baseline and follow‐up could not be assessed in a similar manner due to the absence of some IADL‐related questions in the follow‐up survey. Thus, the effect of time‐varying confounding in the analyses was assessed to be small. Sixth, the generalisability of the findings was limited because JAGES data is not nationally representative. On the other hand, the major strength of this study was that it was the first study to explain to some extent the mechanism of the relationship between oral health and social isolation. In addition, causal mediation analysis was used to estimate the magnitude of the mediated pathways through the three included oral functions, allowing for mediator‐exposure interaction. The findings of this study could be used as a foundation for future causal mediation studies that need to investigate remaining potential oral health‐related mediators (dental pain and discomfort) as well as the potential non‐oral health‐related mediators (depression, social anxiety, chronic stress, reduced self‐esteem and unhealthy diet) for the association between oral health and social isolation.

## 5 Conclusion

Considering the low precise and high uncertain estimates, the findings did not reject the null hypothesis; however, the mediation effect of oral functions varied by oral health status. Compared with those with 20 or more teeth, oral functions might have mediated the association between oral health and social isolation for those with 10–19 teeth with and without dental prosthesis, but might not have for those with 0–9 teeth with and without dental prosthesis. The proportion mediated through having difficulty in chewing was higher than the other two oral functions among those having 10–19 teeth with and without dental prosthesis. These findings suggested the presence of non‐oral health related mediators for the association between oral health and social isolation, especially among those with severe tooth loss (0–9 teeth groups). Further research exploring more mediating pathways using larger datasets to help narrow the confidence interval and provide more precise and significant estimates of the effect is required.

## Author Contributions

Hazem Abbas contributed to the conception of the study, study design, project administration, data acquisition, data analysis, interpretation of the findings and drafted the manuscript; Kenji Takeuchi, Taro Kusama and Sakura Kiuchi contributed to the study design, data acquisition, data analysis and interpretation of the findings; Ken Osaka contributed to the data acquisition. All authors critically revised the manuscript, gave final approval, and agreed to be accountable for all aspects of the work, ensuring integrity and accuracy.

## Conflicts of Interest

The authors declare no conflicts of interest.

## Supporting information


**Table S1:** Baseline characteristics of the participants by oral health status showing the missing data before multiple imputation (*n* = 6103).
**Table S2:** The proportion of incidence of social isolation at follow up showing the missing data before multiple imputation (*n* = 6103).
**Table S3:** The mediating effect of oral functions decline in the association between tooth loss and social isolation after multiple imputation (*n* = 6103).
**Table S4:** The mediating effect of oral functions decline in the association between dental prosthesis use and social isolation after multiple imputation (*n* = 6103).
**Table S5:** Robustness to unmeasured confounding (E‐values) for the total effect (TE) of the association between oral health and social isolation.
**Table S6:** The association between oral health and social isolation at follow‐up after multiple imputation (*n* = 6103).
**Table S7:** The association between oral health and social isolation at follow‐up using complete data analysis.
**Table S8:** The interaction and effect modification between dental prosthesis use and number of remaining teeth on social isolation at follow‐up after multiple imputation (*n* = 6103).
**Table S9:** The association between oral health and social isolation at follow‐up using inverse probability of treatment weighting (IPTW) after multiple imputation (*n* = 6103).

## Data Availability

The data from the Japan Gerontological Evaluation Study (JAGES) is available upon requests submitted to the JAGES data management office. Applications for data usage are accepted online from the JAGES website. https://www.jages.net/.

## References

[1] World Health Organization , “Social Isolation and Loneliness Among Older People: Advocacy Brief,” (2021, accessed July 22, 2024), https://www.who.int/publications/i/item/9789240030749.

[2] World Health Organization , “WHO Commission on Social Connection,” (2025, accessed August 6, 2025), https://www.who.int/groups/commission‐on‐social‐connection.

[3] J. Holt‐Lunstad , T. B. Smith , M. Baker , T. Harris , and D. Stephenson , “Loneliness and Social Isolation as Risk Factors for Mortality: A Meta‐Analytic Review,” Perspectives on Psychological Science 10, no. 2 (2015): 227–237, 10.1177/1745691614568352. 25910392

[4] R. Mistry , J. Rosansky , J. McGuire , C. McDermott , and L. Jarvik , “Social Isolation Predicts Re‐Hospitalization in a Group of Older American Veterans Enrolled in the UPBEAT Program,” International Journal of Geriatric Psychiatry 16, no. 10 (2001): 950–959, 10.1002/gps.447. 11607938

[5] K. A. Faulkner , J. A. Cauley , J. M. Zmuda , J. M. Griffin , and M. C. Nevitt , “Is Social Integration Associated With the Risk of Falling in Older Community‐Dwelling Women?,” Journals of Gerontology. Series A, Biological Sciences and Medical Sciences 58, no. 10 (2003): 954–959, 10.1093/gerona/58.10.m954. 14570865

[6] E. Klinenberg , “Social Isolation, Loneliness, and Living Alone: Identifying the Risks for Public Health,” American Journal of Public Health 106, no. 5 (2016): 786–787, 10.2105/AJPH.2016.303166. 27049414 PMC4985072

[7] E. Courtin and M. Knapp , “Social Isolation, Loneliness and Health in Old Age: A Scoping Review,” Health & Social Care in the Community 25, no. 3 (2017): 799–812, 10.1111/hsc.12311. 26712585

[8] World Health Organization , “Global Health and Aging. Global Health and Aging,” (2011, accessed January 13, 2021), https://www.who.int/ageing/publications/global_health.pdf.

[9] H. Abbas , J. Aida , U. Cooray , et al., “Does Remaining Teeth and Dental Prosthesis Associate With Social Isolation? A Six‐Year Longitudinal Study From the Japan Gerontological Evaluation Study (JAGES),” Community Dentistry and Oral Epidemiology 1 (2022): 1–354, 10.1111/CDOE.12746. 35352849

[10] S. Koyama , M. Saito , N. Cable , et al., “Examining the Associations Between Oral Health and Social Isolation: A Cross‐National Comparative Study Between Japan and England,” Social Science & Medicine 277 (2021): 113895, 10.1016/J.SOCSCIMED.2021.113895. 33882441

[11] S. M. Rodrigues , A. C. Oliveira , A. M. D. Vargas , A. N. Moreira , and E. Ferreira , “Implications of Edentulism on Quality of Life Among Elderly,” International Journal of Environmental Research and Public Health 9, no. 1 (2012): 100–109, 10.3390/ijerph9010100. 22470281 PMC3315080

[12] C. Knipfer , M. Riemann , T. Bocklet , et al., “Speech Intelligibility Enhancement After Maxillary Denture Treatment and Its Impact on Quality of Life,” International Journal of Prosthodontics 27, no. 1 (2014): 61–69, 10.11607/ijp.3597. 24392479

[13] A. J. Hickey and M. Salter , “Prosthodontic and Psychological Factors in Treating Patients With Congenital and Craniofacial Defects,” Journal of Prosthetic Dentistry 95 (2006): 392–396, 10.1016/j.prosdent.2006.03.002. 16679134

[14] Y. Tamada , K. Takeuchi , T. Kusama , et al., “Reduced Number of Teeth With and Without Dental Prostheses and Low Frequency of Laughter in Older Adults: Mediation by Poor Oral Function,” Journal of Prosthodontic Research 68, no. 3 (2024): 441–448, 10.2186/JPR.JPR_D_23_00071. 37793820

[15] R. E. Nowjack‐Raymer and A. Sheiham , “Numbers of Natural Teeth, Diet, and Nutritional Status in US Adults,” Journal of Dental Research 86, no. 12 (2007): 1171–1175, 10.1177/154405910708601206. 18037650

[16] K. Wakai , M. Naito , T. Naito , et al., “Tooth Loss and Intakes of Nutrients and Foods: A Nationwide Survey of Japanese Dentists,” Community Dentistry and Oral Epidemiology 38, no. 1 (2010): 43–49, 10.1111/j.1600-0528.2009.00512.x. 19922495

[17] B. Fougère and J. E. Morley , “Weight Loss Is a Major Cause of Frailty,” Journal of Nutrition, Health & Aging 21, no. 9 (2017): 933–935, 10.1007/s12603-017-0971-7. PMC12879830 29083432

[18] H. C. Roberts , S. E. R. Lim , N. J. Cox , and K. Ibrahim , “The Challenge of Managing Undernutrition in Older People With Frailty,” Nutrients 11, no. 4 (2019): 808, 10.3390/nu11040808. 30974825 PMC6521101

[19] H. Abbas , J. Aida , S. Kiuchi , K. Kondo , and K. Osaka , “Oral Status and Homebound Status: A 6‐Year Bidirectional Exploratory Prospective Cohort Study,” Oral Diseases 29, no. 3 (2023): 1291–1298, 10.1111/ODI.14039. 34601759

[20] Y. Shimazaki , M. Saito , T. Nonoyama , and Y. Inamoto , “Validity of the Self‐Reported Number of Teeth in Independent Older People in Japan,” BMC Geriatrics 24, no. 1 (2024): 900, 10.1186/S12877-024-05512-1. 39482622 PMC11526520

[21] M. Ueno , T. Shimazu , N. Sawada , S. Tsugane , and Y. Kawaguchi , “Validity of Self‐Reported Tooth Counts and Masticatory Status Study of a Japanese Adult Population,” Journal of Oral Rehabilitation 45, no. 5 (2018): 393–398, 10.1111/joor.12615. 29420835

[22] O. A. Fakoya , N. K. McCorry , and M. Donnelly , “Loneliness and Social Isolation Interventions for Older Adults: A Scoping Review of Reviews,” BMC Public Health 20, no. 1 (2020): 129, 10.1186/s12889-020-8251-6. 32054474 PMC7020371

[23] N. R. Nicholson , “A Review of Social Isolation: An Important but Underassessed Condition in Older Adults,” Journal of Primary Prevention 33, no. 2 (2012): 137–152, 10.1007/s10935-012-0271-2. 22766606

[24] L. Grenade , D. Boldy , and R. Fellow , “Social Isolation and Loneliness Among Older People: Issues and Future Challenges in Community and Residential Settings,” Australian Health Review 32, no. 3 (2008): 468–478. 18666874 10.1071/ah080468

[25] N. R. Nicholson , “Social Isolation in Older Adults: An Evolutionary Concept Analysis,” Journal of Advanced Nursing 65, no. 6 (2009): 1342–1352, 10.1111/j.1365-2648.2008.04959.x. 19291185

[26] T. Tsuji , M. Saito , T. Ikeda , et al., “Change in the Prevalence of Social Isolation Among the Older Population From 2010 to 2016: A Repeated Cross‐Sectional Comparative Study of Japan and England,” Archives of Gerontology and Geriatrics 91 (2020): 104237, 10.1016/j.archger.2020.104237. 32861955

[27] T. Ikeda , N. Cable , M. Saito , et al., “Association Between Social Isolation and Smoking in Japan and England,” Journal of Epidemiology 31 (2020): 523–529, 10.2188/jea.je20200138. PMC8421201 32779628

[28] M. Saito , J. Aida , N. Cable , et al., “Cross‐National Comparison of Social Isolation and Mortality Among Older Adults: A 10‐Year Follow‐Up Study in Japan and England,” Geriatrics & Gerontology International 21 (2020): 209–214, 10.1111/ggi.14118. 33350047 PMC7898799

[29] T. Noguchi , M. Saito , J. Aida , and N. Cable , “Association Between Social Isolation and Depression Onset Among Older Adults: A National Longitudinal Study in England and Japan,” BMJ Open 11, no. 3 (2021): 45834, 10.1136/bmjopen-2020-045834. PMC7978252 33737442

[30] H. Abbas , J. Aida , M. Saito , et al., “Income or Education, Which Has a Stronger Association With Dental Implant Use in Elderly People in Japan?,” International Dental Journal 69, no. 6 (2019): 454–462, 10.1111/idj.12491. 31250446 PMC9378978

[31] W. Koyano , H. Shibata , K. Nakazato , H. Haga , and Y. Suyama , “Measurement of Competence: Reliability and Validity of the TMIG Index of Competence,” Archives of Gerontology and Geriatrics 13, no. 2 (1991): 103–116, 10.1016/0167-4943(91)90053-S. 15374421

[32] “Statistics Bureau Ministry of Internal Affairs and Communications Japan,” Statistical Handbook of Japan 2010 (2010).

[33] W. J. Burke , W. H. Roccaforte , and S. P. Wengel , “The Short Form of the Geriatric Depression Scale: A Comparison With the 30‐Item Form,” Journal of Geriatric Psychiatry and Neurology 4, no. 3 (1991): 173–178, 10.1177/089198879100400310. 1953971

[34] C. Murata , K. Kondo , H. Hirai , Y. Ichida , and T. Ojima , “Association Between Depression and Socio‐Economic Status Among Community‐Dwelling Elderly in Japan: The Aichi Gerontological Evaluation Study (AGES),” Health & Place 14, no. 3 (2008): 406–414, 10.1016/j.healthplace.2007.08.007. 17913562

[35] L. Valeri and T. J. VanderWeele , “Mediation Analysis Allowing for Exposure‐Mediator Interactions and Causal Interpretation: Theoretical Assumptions and Implementation With SAS and SPSS Macros,” Psychological Methods 18, no. 2 (2013): 137–150, 10.1037/A0031034. 23379553 PMC3659198

[36] T. J. Van Der Weele and P. Ding , “Sensitivity Analysis in Observational Research: Introducing the E‐Value,” Annals of Internal Medicine 167, no. 4 (2017): 268–274, 10.7326/M16-2607. 28693043

[37] Y. Matsuyama , H. Jürges , M. Dewey , and S. Listl , “Causal Effect of Tooth Loss on Depression: Evidence From a Population‐Wide Natural Experiment in the USA,” Epidemiology and Psychiatric Sciences 30 (2021): e38, 10.1017/S2045796021000287. 34030762 PMC8157508

[38] T. Elmer and C. Stadtfeld , “Depressive Symptoms Are Associated With Social Isolation in Face‐to‐Face Interaction Networks,” Scientific Reports 10, no. 1 (2020): 1–12, 10.1038/s41598-020-58297-9. 31996728 PMC6989520

[39] S. Koka and A. Gupta , “Association Between Missing Tooth Count and Mortality: A Systematic Review,” Journal of Prosthodontic Research 62, no. 2 (2018): 134–151, 10.1016/j.jpor.2017.08.003. 28869174

[40] S. Shirahase , “Income Inequality Among Older People in Rapidly Aging Japan,” Research in Social Stratification and Mobility 41 (2015): 1–10, 10.1016/J.RSSM.2015.03.001.

[41] K. Seiyama , “Income Disparity Among the Elderly in Japan: Life‐Course Factors Affecting Retired Life,” IASR Working Paper Series. Kwansei Gakuin University (2016).

