# Supplementary material for: Oral Health and Social Isolation After 6 Years: Mediation of Oral Functions
Source: Community Dent Oral Epidemiol. 2025 Oct 26;54(2):220–30. doi: 10.1111/cdoe.70035 (PMC13000996; doi:10.1111/cdoe.70035)
Supplement: Supplementary file 1 — Table S1: Baseline characteristics of the participants by oral health status showing the missing data before multiple imputation (n = 6103). Table S2: The proportion of incidence of social isolation at follow up showing the missing data before multiple imputation (n = 6103). Table S3: The mediating effect of oral functions decline in the association between tooth loss and social isolation after multiple imputation (n = 6103). Table S4: The mediating effect of oral functions decline in the association between dental prosthesis use and social isolation after multiple imputation (n = 6103). Table S5: Robustness to unmeasured confounding (E‐values) for the total effect (TE) of the association between oral health and social isolation. Table S6: The association between oral health and social isolation at follow‐up after multiple imputation (n = 6103). Table S7: The association between oral health and social isolation at follow‐up using complete data analysis. Table S8: The interaction and effect modification between dental prosthesis use and number of remaining teeth on social isolation at follow‐up after multiple imputation (n = 6103). Table S9: The association between oral health and social isolation at follow‐up using inverse probability of treatment weighting (IPTW) after multiple imputation (n = 6103). [file CDOE-54-220-s001.docx]

**Supplementary Table 1. Baseline characteristics of the participants by oral health status showing the missing data before multiple imputation (n=6,103).**

|  | **Total** | | **≥20 teeth** | | **10-19 teeth with dental prosthesis** | | **10-19 teeth without dental prosthesis** | | **0-9 teeth with dental prosthesis** | | **0-9 teeth without dental prosthesis** | | **Missing** | |
| --- | --- | --- | --- | --- | --- | --- | --- | --- | --- | --- | --- | --- | --- | --- |
|  | **No.** | **%** | **No.** | **%** | **No.** | **%** | **No.** | **%** | **No.** | **%** | **No.** | **%** | **No.** | **%** |
| **Difficulty in speaking** |  |  |  |  |  |  |  |  |  |  |  |  |  |  |
| No | 5299 | 86.8 | 2117 | 40.0 | 863 | 16.3 | 486 | 9.2 | 1114 | 21.0 | 418 | 7.9 | 301 | 5.7 |
| Yes | 321 | 5.3 | 23 | 7.2 | 66 | 20.6 | 15 | 4.7 | 137 | 42.7 | 53 | 16.5 | 27 | 8.4 |
| Missing | 483 | 7.9 | 186 | 38.5 | 61 | 12.6 | 57 | 11.8 | 83 | 17.2 | 55 | 11.4 | 41 | 8.5 |
| **Problems in smiling** |  |  |  |  |  |  |  |  |  |  |  |  |  |  |
| No | 5289 | 86.7 | 2105 | 39.8 | 864 | 16.3 | 473 | 8.9 | 1123 | 21.2 | 420 | 7.9 | 304 | 5.7 |
| Yes | 331 | 5.4 | 35 | 10.6 | 65 | 19.6 | 28 | 8.5 | 128 | 38.7 | 51 | 15.4 | 24 | 7.3 |
| Missing | 483 | 7.9 | 186 | 38.5 | 61 | 12.6 | 57 | 11.8 | 83 | 17.2 | 55 | 11.4 | 41 | 8.5 |
| **Difficulty in chewing** |  |  |  |  |  |  |  |  |  |  |  |  |  |  |
| No | 5592 | 91.6 | 2270 | 40.6 | 923 | 16.5 | 519 | 9.3 | 1140 | 20.4 | 414 | 7.4 | 326 | 5.8 |
| Yes | 397 | 6.5 | 18 | 4.5 | 54 | 13.6 | 28 | 7.1 | 166 | 41.8 | 97 | 24.4 | 34 | 8.6 |
| Missing | 114 | 1.9 | 38 | 33.3 | 13 | 11.4 | 11 | 9.6 | 28 | 24.6 | 15 | 13.2 | 9 | 7.9 |
| **Age** |  |  |  |  |  |  |  |  |  |  |  |  |  |  |
| 65-69 Years | 2115 | 34.7 | 979 | 46.3 | 382 | 18.1 | 202 | 9.6 | 354 | 16.7 | 122 | 5.8 | 76 | 3.6 |
| 70-74 Years | 2104 | 34.5 | 834 | 39.6 | 348 | 16.5 | 199 | 9.5 | 438 | 20.8 | 166 | 7.9 | 119 | 5.7 |
| 75-79 Years | 1249 | 20.5 | 382 | 30.6 | 171 | 13.7 | 119 | 9.5 | 310 | 24.8 | 153 | 12.2 | 114 | 9.1 |
| 80-84 Years | 510 | 8.4 | 114 | 22.4 | 78 | 15.3 | 31 | 6.1 | 172 | 33.7 | 68 | 13.3 | 47 | 9.2 |
| ≥85 Years | 125 | 2.0 | 17 | 13.6 | 11 | 8.8 | 7 | 5.6 | 60 | 48.0 | 17 | 13.6 | 13 | 10.4 |
| **Sex** |  |  |  |  |  |  |  |  |  |  |  |  |  |  |
| Male | 2727 | 44.7 | 1070 | 39.2 | 476 | 17.5 | 241 | 8.8 | 596 | 21.9 | 232 | 8.5 | 112 | 4.1 |
| Female | 3376 | 55.3 | 1256 | 37.2 | 514 | 15.2 | 317 | 9.4 | 738 | 21.9 | 294 | 8.7 | 257 | 7.6 |
| **Years of formal Education** |  |  |  |  |  |  |  |  |  |  |  |  |  |  |
| ≥13 years | 1111 | 18.2 | 542 | 48.8 | 203 | 18.3 | 71 | 6.4 | 199 | 17.9 | 49 | 4.4 | 47 | 4.2 |
| 10-12 years | 2221 | 36.4 | 917 | 41.3 | 404 | 18.2 | 217 | 9.8 | 473 | 21.3 | 132 | 5.9 | 78 | 3.5 |
| ≤9 years | 2684 | 44.0 | 839 | 31.3 | 377 | 14.0 | 265 | 9.9 | 640 | 23.8 | 334 | 12.4 | 229 | 8.5 |
| Missing | 87 | 1.4 | 28 | 32.2 | 6 | 6.9 | 5 | 5.7 | 22 | 25.3 | 11 | 12.6 | 15 | 17.2 |
| **Equivalised income level** |  |  |  |  |  |  |  |  |  |  |  |  |  |  |
| <20,000 USD/Year | 2397 | 39.3 | 825 | 34.4 | 357 | 14.9 | 233 | 9.7 | 552 | 23.0 | 270 | 11.3 | 160 | 6.7 |
| 20,000-39,999 USD/Year | 2235 | 36.6 | 960 | 43.0 | 415 | 18.6 | 197 | 8.8 | 455 | 20.4 | 124 | 5.5 | 84 | 3.8 |
| >40,000 USD/Year | 612 | 10.0 | 300 | 49.0 | 109 | 17.8 | 34 | 5.6 | 116 | 19.0 | 29 | 4.7 | 24 | 3.9 |
| Missing | 859 | 14.1 | 241 | 28.1 | 109 | 12.7 | 94 | 10.9 | 211 | 24.6 | 103 | 12.0 | 101 | 11.8 |
| **Independence in activities of daily living (IADL)** |  |  |  |  |  |  |  |  |  |  |  |  |  |  |
| IADL score 0-12 (dependent) | 2741 | 44.9 | 997 | 36.4 | 429 | 15.7 | 276 | 10.1 | 625 | 22.8 | 274 | 10.0 | 140 | 5.1 |
| IADL score 13 (independent) | 2802 | 45.9 | 1167 | 41.6 | 489 | 17.5 | 236 | 8.4 | 575 | 20.5 | 203 | 7.2 | 132 | 4.7 |
| Missing | 560 | 9.2 | 162 | 28.9 | 72 | 12.9 | 46 | 8.2 | 134 | 23.9 | 49 | 8.8 | 97 | 17.3 |
| **Living area** |  |  |  |  |  |  |  |  |  |  |  |  |  |  |
| Urban | 1211 | 19.8 | 524 | 43.3 | 195 | 16.1 | 105 | 8.7 | 225 | 18.6 | 56 | 4.6 | 106 | 8.8 |
| Sub-urban | 2424 | 39.7 | 991 | 40.9 | 408 | 16.8 | 214 | 8.8 | 524 | 21.6 | 182 | 7.5 | 105 | 4.3 |
| Rural | 2468 | 40.4 | 811 | 32.9 | 387 | 15.7 | 239 | 9.7 | 585 | 23.7 | 288 | 11.7 | 158 | 6.4 |
| **Depressive symptoms (GDS-15)** |  |  |  |  |  |  |  |  |  |  |  |  |  |  |
| No (0-4) | 4069 | 66.7 | 1646 | 40.5 | 683 | 16.8 | 374 | 9.2 | 856 | 21.0 | 319 | 7.8 | 191 | 4.7 |
| Yes (≥5) | 1207 | 19.8 | 400 | 33.1 | 187 | 15.5 | 123 | 10.2 | 293 | 24.3 | 125 | 10.4 | 79 | 6.5 |
| Missing | 827 | 13.6 | 280 | 33.9 | 120 | 14.5 | 61 | 7.4 | 185 | 22.4 | 82 | 9.9 | 99 | 12.0 |
| **Total** | 6103 | 100.0 | 2326 | 38.1 | 990 | 16.2 | 558 | 9.1 | 1334 | 21.9 | 526 | 8.6 | 369 | 6.0 |

**Supplementary Table 2. The proportion of incidence of social isolation at follow up showing the missing data before multiple imputation (n=6,103).**

|  | **Total** | | **no isolation** | | **isolated** | | **Missing** | |
| --- | --- | --- | --- | --- | --- | --- | --- | --- |
|  | **No.** | **%** | **No.** | **%** | **No.** | **%** | **No.** | **%** |
| **Oral health status** |  |  |  |  |  |  |  |  |
| ≥20 teeth | 2326 | 38.1 | 2005 | 86.2 | 55 | 2.4 | 266 | 11.4 |
| 10-19 teeth with dental prosthesis | 990 | 16.2 | 833 | 84.1 | 26 | 2.6 | 131 | 13.2 |
| 10-19 teeth without dental prosthesis | 558 | 9.1 | 461 | 82.6 | 16 | 2.9 | 81 | 14.5 |
| 0-9 teeth with dental prosthesis | 1334 | 21.9 | 1077 | 80.7 | 39 | 2.9 | 218 | 16.3 |
| 0-9 teeth without dental prosthesis | 526 | 8.6 | 389 | 74.0 | 29 | 5.5 | 108 | 20.5 |
| Missing | 369 | 6.0 | 265 | 71.8 | 17 | 4.6 | 87 | 23.6 |
| **Difficulty in speaking** |  |  |  |  |  |  |  |  |
| No | 5299 | 86.8 | 4399 | 83.0 | 156 | 2.9 | 744 | 14.0 |
| Yes | 321 | 5.3 | 263 | 81.9 | 11 | 3.4 | 47 | 14.6 |
| Missing | 483 | 7.9 | 368 | 76.2 | 15 | 3.1 | 100 | 20.7 |
| **Problems in smiling** |  |  |  |  |  |  |  |  |
| No | 5289 | 86.7 | 4391 | 83.0 | 154 | 2.9 | 744 | 14.1 |
| Yes | 331 | 5.4 | 271 | 81.9 | 13 | 3.9 | 47 | 14.2 |
| Missing | 483 | 7.9 | 368 | 76.2 | 15 | 3.1 | 100 | 20.7 |
| **Difficulty in chewing** |  |  |  |  |  |  |  |  |
| No | 5592 | 91.6 | 4648 | 83.1 | 155 | 2.8 | 789 | 14.1 |
| Yes | 397 | 6.5 | 295 | 74.3 | 22 | 5.5 | 80 | 20.2 |
| Missing | 114 | 1.9 | 87 | 76.3 | 5 | 4.4 | 22 | 19.3 |
| **Age** |  |  |  |  |  |  |  |  |
| 65-69 Years | 2115 | 34.7 | 1834 | 86.7 | 53 | 2.5 | 228 | 10.8 |
| 70-74 Years | 2104 | 34.5 | 1743 | 82.8 | 56 | 2.7 | 305 | 14.5 |
| 75-79 Years | 1249 | 20.5 | 981 | 78.5 | 46 | 3.7 | 222 | 17.8 |
| 80-84 Years | 510 | 8.4 | 379 | 74.3 | 21 | 4.1 | 110 | 21.6 |
| ≥85 Years | 125 | 2.0 | 93 | 74.4 | 6 | 4.8 | 26 | 20.8 |
| **Sex** |  |  |  |  |  |  |  |  |
| Male | 2727 | 44.7 | 2280 | 83.6 | 108 | 4.0 | 339 | 12.4 |
| Female | 3376 | 55.3 | 2750 | 81.5 | 74 | 2.2 | 552 | 16.4 |
| **Years of formal Education** |  |  |  |  |  |  |  |  |
| ≥13 years | 1111 | 18.2 | 991 | 89.2 | 29 | 2.6 | 91 | 8.2 |
| 10-12 years | 2221 | 36.4 | 1905 | 85.8 | 65 | 2.9 | 251 | 11.3 |
| ≤9 years | 2684 | 44.0 | 2076 | 77.3 | 83 | 3.1 | 525 | 19.6 |
| Missing | 87 | 1.4 | 58 | 66.7 | 5 | 5.7 | 24 | 27.6 |
| **Equivalised income level** |  |  |  |  |  |  |  |  |
| <20,000 USD/Year | 2397 | 39.3 | 1905 | 79.5 | 94 | 3.9 | 398 | 16.6 |
| 20,000-39,999 USD/Year | 2235 | 36.6 | 1946 | 87.1 | 59 | 2.6 | 230 | 10.3 |
| >40,000 USD/Year | 612 | 10.0 | 540 | 88.2 | 7 | 1.1 | 65 | 10.6 |
| Missing | 859 | 14.1 | 639 | 74.4 | 22 | 2.6 | 198 | 23.1 |
| **Independence in activities of daily living (IADL)** |  |  |  |  |  |  |  |  |
| IADL score 0-12 (dependent) | 2741 | 44.9 | 2171 | 79.2 | 121 | 4.4 | 449 | 16.4 |
| IADL score 13 (independent) | 2802 | 45.9 | 2447 | 87.3 | 33 | 1.2 | 322 | 11.5 |
| Missing | 560 | 9.2 | 412 | 73.6 | 28 | 5.0 | 120 | 21.4 |
| **Living area** |  |  |  |  |  |  |  |  |
| Urban | 1211 | 19.8 | 1020 | 84.2 | 53 | 4.4 | 138 | 11.4 |
| Sub-urban | 2424 | 39.7 | 2011 | 83.0 | 64 | 2.6 | 349 | 14.4 |
| Rural | 2468 | 40.4 | 1999 | 81.0 | 65 | 2.6 | 404 | 16.4 |
| **Depressive symptoms (GDS-15)** |  |  |  |  |  |  |  |  |
| No (0-4) | 4069 | 66.7 | 3466 | 85.2 | 90 | 2.2 | 513 | 12.6 |
| Yes (≥5) | 1207 | 19.8 | 940 | 77.9 | 62 | 5.1 | 205 | 17.0 |
| Missing | 827 | 13.6 | 624 | 75.5 | 30 | 3.6 | 173 | 20.9 |
| **Total** | 6103 | 100.0 | 5030 | 82.4 | 182 | 3.0 | 891 | 14.6 |

GDS=Geriatric depression scale.

**Supplementary Table 3. The mediating effect of oral functions decline in the association between tooth loss and social isolation after multiple imputation (n=6,103)**

|  | **Mediators** | | | | | | | | |
| --- | --- | --- | --- | --- | --- | --- | --- | --- | --- |
|  | **Difficulty in speaking** | | | **Problems in smiling** | | | **Difficulty in chewing** | | |
| Number of remaining teeth (ref. ≥20) | **OR** | (95%CI) | | **OR** | (95%CI) | | **OR** | (95%CI) | |
| **10-19 teeth** |  | | | | | | | | |
| **Natural direct effect** | **1.17** | 0.73 | 1.60 | **1.17** | 0.73 | 1.60 | **1.09** | 0.66 | 1.52 |
| **Natural indirect effect** | **1.01** | 0.96 | 1.07 | **1.02** | 0.96 | 1.08 | **1.05** | 0.98 | 1.13 |
| **Total effect** | **1.18** | 0.75 | 1.61 | **1.19** | 0.76 | 1.62 | **1.15** | 0.72 | 1.58 |
| **Proportion mediated** | **6.4%** | | | **12.1%** | | | **37.7%** | | |
| **0-9 teeth** |  | | | | | | | | |
| **Natural direct effect** | **1.48** | 1.04 | 1.93 | **1.43** | 0.98 | 1.88 | **1.44** | 0.99 | 1.90 |
| **Natural indirect effect** | **0.97** | 0.92 | 1.02 | **0.99** | 0.93 | 1.06 | **0.99** | 0.92 | 1.06 |
| **Total effect** | **1.43** | 0.99 | 1.87 | **1.42** | 0.98 | 1.86 | **1.43** | 0.99 | 1.87 |
| **Proportion mediated** | **-** | | | **-** | | | **-** | | |

*Note 1*: OR = odds ratio, 95%CI = 95% confidence intervals.

*Note 2*: Adjusted for age, sex, educational attainment, income, activities of daily living, living area and having depressive symptoms.

**Supplementary Table 4. The mediating effect of oral functions decline in the association between dental prosthesis use and social isolation after multiple imputation (n=6,103)**

| **Dental prosthesis use** | **Mediators** | | | | | | | | |
| --- | --- | --- | --- | --- | --- | --- | --- | --- | --- |
|  | **Difficulty in speaking** | | | **Problems in smiling** | | | **Difficulty in chewing** | | |
| **(ref. use dental prosthesis)** | **OR** | (95%CI) | | **OR** | (95%CI) | | **OR** | (95%CI) | |
| **No dental prosthesis use** |  | | | | | | | | |
| **Natural direct effect** | 0.84 | 0.53 | 1.16 | 0.85 | 0.53 | 1.17 | 0.84 | 0.52 | 1.16 |
| **Natural indirect effect** | 1.00 | 0.97 | 1.04 | 1.00 | 0.97 | 1.03 | 1.01 | 0.99 | 1.03 |
| **Total effect** | 0.84 | 0.53 | 1.16 | 0.85 | 0.53 | 1.17 | 0.85 | 0.53 | 1.17 |
| **Proportion mediated** | **-** | | | **-** | | | **-** | | |

*Note 1*: OR = odds ratio, 95%CI = 95% confidence intervals.

*Note 2*: Adjusted for age, sex, educational attainment, income, activities of daily living, living area and having depressive symptoms.

**Supplementary Table 5. Robustness to unmeasured confounding (E-values) for the total effect (TE) of the association between oral health and social isolation.**

|  | **Mediators** | | | | | |
| --- | --- | --- | --- | --- | --- | --- |
|  | **Difficulty in speaking** | | **Problems in smiling** | | **Difficulty in chewing** | |
| Number of remaining teeth (ref. ≥20) | **E-value for point estimate*** | **E-value for lower confidence limit**** | **E-value for point estimate*** | **E-value for lower confidence limit**** | **E-value for point estimate*** | **E-value for lower confidence limit**** |
| **10-19 with dental prosthesis** |  | | | | | |
| **Total effect** | **1.46** | 1.00 | **1.49** | 1.00 | **1.37** | 1.00 |
| **10-19 without dental prosthesis** |  | | | | | |
| **Total effect** | **1.57** | 1.00 | **1.59** | 1.00 | **1.57** | 1.00 |
| **0-9 with dental prosthesis** |  | | | | | |
| **Total effect** | **1.62** | 1.00 | **1.59** | 1.00 | **1.62** | 1.00 |
| **0-9 without dental prosthesis** |  | | | | | |
| **Total effect** | **3.97** | 1.95 | **3.82** | 1.83 | **4.11** | 2.26 |

Adjusted for age, sex, educational attainment, income, activities of daily living, living area and having depressive symptoms.

***** E-values present the minimum strength of association on the odds ratio scale that an unmeasured confounder would need to have with both exposure and outcome to fully explain away the observed association conditional on included confounders.

****** E-values of the 95% confidence interval limit closest to the null denotes the minimum strength of association on the odds ratio scale that an unmeasured confounder would need to have with both exposure and the outcome to shift the 95% confidence interval to include the null value conditional on included confounders.

Abbreviations: ref., reference

**Supplementary Table 6. The association between oral health and social isolation at follow-up after multiple imputation (n=6,103)**

|  | **Crude model** | | | **Adjusted model^†^** | | |  |
| --- | --- | --- | --- | --- | --- | --- | --- |
|  | **Odds ratio** | **95% CI** | | **Odds ratio** | **95% CI** | |  |
|  |  |  |  |  |  |  |  |
| **Number of teeth & dental prosthesis use** |  |  |  |  |  |  |  |
| **≥20 teeth** | **1.00** |  |  | **1.00** |  |  |  |
| **10-19 teeth with dental prosthesis** | **1.12** | 0.68 | 1.84 | **1.08** | 0.65 | 1.80 |  |
| **10-19 teeth without dental prosthesis** | **1.23** | 0.67 | 2.24 | **1.06** | 0.57 | 1.99 |  |
| **0-9 teeth with dental prosthesis** | **1.31** | 0.85 | 2.01 | **1.09** | 0.70 | 1.71 |  |
| **0-9 teeth without dental prosthesis** | **2.60*** | 1.67 | 4.06 | **2.05*** | 1.27 | 3.28 |  |

†The adjusted model was adjusted for age, sex, educational attainment, equivalised income level, independence in activities of daily living, living area, and having depressive symptoms measured by the geriatric depression scale (GDS-15).

Abbreviations; CI= Confidence interval

* p-value <0.05

**Supplementary Table 7. The association between oral health and social isolation at follow-up using complete data analysis.**

|  | **Crude model**  **(n=4,930)** | | | **Adjusted model^†^**  **(n=3,625)** | | |  |
| --- | --- | --- | --- | --- | --- | --- | --- |
|  | **Odds ratio** | **95% CI** | | **Odds ratio** | **95% CI** | |  |
|  |  |  |  |  |  |  |  |
| **Number of teeth & dental prosthesis use** |  |  |  |  |  |  |  |
| **≥20 teeth** | **1.00** |  |  | **1.00** |  |  |  |
| **10-19 teeth with dental prosthesis** | **1.14** | 0.71 | 1.83 | **0.96** | 0.52 | 1.78 |  |
| **10-19 teeth without dental prosthesis** | **1.27** | 0.72 | 2.23 | **1.53** | 0.79 | 2.96 |  |
| **0-9 teeth with dental prosthesis** | **1.32** | 0.87 | 2.00 | **1.35** | 0.80 | 2.28 |  |
| **0-9 teeth without dental prosthesis** | **2.72*** | 1.71 | 4.32 | **2.23*** | 1.21 | 4.14 |  |

†The adjusted model was adjusted for age, sex, educational attainment, equivalised income level, independence in activities of daily living, living area, and having depressive symptoms measured by the geriatric depression scale (GDS-15).

Abbreviations; CI= Confidence interval

* p-value <0.05

**Supplementary Table 8. The interaction and effect modification between dental prosthesis use and number of remaining teeth on social isolation at follow-up after multiple imputation (n=6,103).**

|  |  | Number of teeth | | | | | | ORs (95%CI) for 10-19 teeth within strata of dental prosthesis use | ORs (95%CI) for 0-9 teeth within strata of dental prosthesis use |
| --- | --- | --- | --- | --- | --- | --- | --- | --- | --- |
|  |  | ≥20 teeth | | 10-19 teeth | | 0-9 teeth | |  |  |
|  |  | N with/with- out social isolation | OR (95%CI) | N with/with- out social isolation | OR (95%CI) | N with/with- out social isolation | OR (95%CI) |  |  |
| Dental prosthesis use | Yes | N/A | 1 | 34/1016 | 1.08 (0.65-1.80); p>0.05 | 54/1378 | 1.09 (0.70-1.71); p>0.05 | 1.29 (0.64-2.59); p>0.05 | 1.24 (0.62-2.46); p>0.05 |
|  | No | N/A | 1 | 21/575 | 1.06 (0.57-1.99); p>0.05 | 41/530 | 2.05 (1.27-3.28); p<0.05 | 1.16 (0.63-2.13); p>0.05 | 2.10 (1.26-3.49); p<0.05 |
| ORs (95%CI) for dental prosthesis use within strata of number of teeth | |  | N/A |  | 0.97 (0.49-1.92); p>0.05 |  | 0.56 (0.35-0.90); p<0.05 |  |  |
| Measure of interaction on additive scale: Synergy index (95%CI) | | | | 0.75 (0.23-1.44); p>0.05 | | 11.67 (0.56-16.72); p>0.05 | |  |  |
| Measure of interaction on multiplicative scale: Ratio of ORs (95%CI) | | | | 0.98 (0.11-2.92); p>0.05 | | 1.88 (0.33-6.32); p>0.05 | |  |  |
| ORs are adjusted for age, sex, educational attainment, income, activities of daily living, living area and having depressive symptoms. | | | | | | | | | |

Abbreviations; OR= odds ratio, CI= Confidence interval

**Supplementary Table 9. The association between oral health and social isolation at follow-up using inverse probability of treatment weighting (IPTW) after multiple imputation (n=6,103).**

|  | **Crude model** | | | **Adjusted model^†^** | | |  |
| --- | --- | --- | --- | --- | --- | --- | --- |
|  | **Coef. **** | **95% CI** | | **Coef. **** | **95% CI** | |  |
|  |  |  |  |  |  |  |  |
| **Number of teeth & dental prosthesis use** |  |  |  |  |  |  |  |
| **≥20 teeth** | **Ref** |  |  | **Ref** |  |  |  |
| **10-19 teeth with dental prosthesis** | **0.00** | -0.01 | 0.02 | **0.01** | -0.01 | 0.02 |  |
| **10-19 teeth without dental prosthesis** | **0.01** | -0.01 | 0.03 | **0.00** | -0.02 | 0.02 |  |
| **0-9 teeth with dental prosthesis** | **0.01** | 0.00 | 0.02 | **0.00** | -0.01 | 0.02 |  |
| **0-9 teeth without dental prosthesis** | **0.04*** | 0.01 | 0.06 | **0.02*** | 0.00 | 0.05 |  |

****** Note 1: the Coef. represented the Average Treatment Effect (ATE) presented as a risk difference on an additive scale.

†The adjusted model was adjusted for age, sex, educational attainment, equivalised income level, independence in activities of daily living, living area, and having depressive symptoms measured by the geriatric depression scale (GDS-15).

Abbreviations; Coef.= Coefficient, CI= Confidence interval, Ref= Reference.

* p-value <0.05
